# Central Blockade of Cannabinoid CB1 and CB2 Receptors Increases Force Generation in Reserpine-Treated Mice, but Only CB1 Blockade Extends the Duration of Effort

**DOI:** 10.1021/acsptsci.6c00206

**Published:** 2026-09-29

**Authors:** Beatriz Carminati dos Santos, Izabely Guedes da Rosa, Laíze de Freitas Mazzardo, Naiara de Souza Santos, Izabel Feltrin Fabro, Tatyana Nery, Amanda Leite Bastos Pereira, Aderbal S. Aguiar Jr

**Affiliations:** † Laboratory of Exercise Biology (LABIOEX), Department of Physical Therapy, Centro de Ciências, Tecnologias e Saúde, Federal University of Santa Catarina, Rua Pedro João Pereira 158, Centro Cívico, Araranguá, Santa Catarina 88905-120, Brazil; ‡ Instituto de Ensino e Pesquisa Remederi, Via Anchieta 1616, Vila Moinho Velho, São Paulo, São Paulo 04246-002, Brazil

**Keywords:** endocannabinoid system, CB1 receptor, CB2 receptor, reserpine, basal ganglia, motor output, grip force, catalepsy

## Abstract

Motor impairment in parkinsonism extends beyond the classical triad to weakness and a reduced capacity to sustain motor output, whose neuromodulatory control is poorly resolved. The endocannabinoid system is a candidate regulator, but CB1 and CB2 receptors have not been separated within one preparation. Reserpine-treated adult female Swiss mice received vehicle, the CB1 antagonist AM251, or the CB2 antagonist AM630 into the lateral ventricle; catalepsy was recorded across three trials and neuromuscular performance in a grip force task. Both antagonists increased the force generated at task onset, by amounts these data cannot distinguish: AM630 by 53.9 gf (95% CI 22.5–85.3) and AM251 by 41.2 gf (95% CI 8.8–73.6). They separated on duration. AM251 increased grip time by 619 ms (95% CI 113–1125), whereas AM630 did not (−70 ms, 95% CI −560 to +421). An interaction contrast on the force–time balance followed from that difference: AM630 shifted the balance toward force (−2.03 SD, *P* = 0.008), AM251 did not (−0.36 SD, *P* = 0.66), and the two differed from each other by 1.67 SD (*P* = 0.022). AM251 also reduced the odds of a cataleptic response (OR 0.16, 95% CI 0.03–0.89). Adjusting for body mass left both force effects unchanged. The dissociation is therefore in the duration of effort rather than in its force: both antagonists raise force, and only CB1 blockade also extends the time over which it is maintained. Delivered intracerebroventricularly, these effects are central in origin but not anatomically localized.

## Graphical abstract

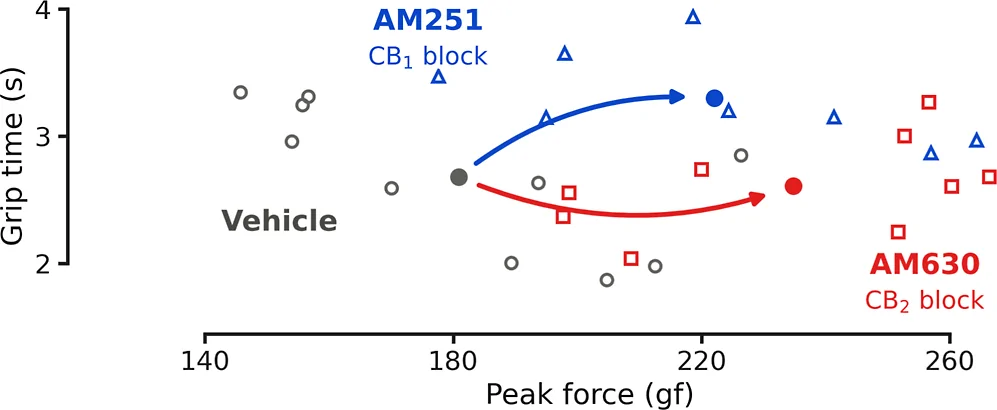

Motor impairment in Parkinson’s disease extends beyond bradykinesia, rigidity and tremor to include weakness and a reduced capacity to sustain effort, and these components limit functional capacity in ways the classical triad does not capture. The willingness to exert effort is itself under neuromodulatory control and is dissociable from hedonic reactivity and unconditioned reinforcement, although motivational and motor systems are closely intertwined rather than independent.^1^ Understanding how they are controlled requires a preparation in which motor output can be measured continuously rather than scored, and a set of pharmacological tools that act within the central nervous system.

Reserpine inhibits the vesicular monoamine transporter 2 and depletes monoamine storage in synaptic vesicles, producing a reversible parkinsonian state.^2^ The construct validity of the model is disputed. Duty and Jenner conclude that it shows little construct validity while retaining sound face validity and strong predictive validity, and note that the model produces no nigral dopaminergic degeneration and is therefore restricted to symptomatic questions.^2^ Leão and colleagues contest that verdict, conceding the absence of demonstrated neurodegeneration and protein aggregation but arguing that the breadth of neurochemical and behavioral features the model reproduces should not be discounted on that basis.^3^ We use the model for what both accounts support: a reversible depleted state suited to mechanistic and symptomatic questions. Peripherally administered at 1–10 mg/kg, reserpine produces a 70–95% depletion of monoamine content across brain regions, together with akinesia, rigidity and catalepsy; at the lower end of that range, which is the dose used here, reported striatal depletion at 24 h is closer to 55%.^3^

This laboratory has characterized a fatigue phenotype in that preparation. Two injections of reserpine at 1 mg/kg reduce voluntary running wheel distance by up to 85% and markedly impair incremental treadmill performance, with lower blood lactate that the authors read as consistent with the reduced workload achieved; the evidence against a peripheral locus in that study came instead from myotube respirometry and cardiac recording.^4^ A single injection at the same dose, 24 h before testing, produces a grip force deficit in the apparatus used here.^5^ Neuromodulation determines motor persistence: in naive mice the ergogenic effect of caffeine requires neuronal adenosine A2A receptors,^6^ and in naive mice both systemic caffeine^7,8^ and the selective A2A antagonist SCH58261 ^8^ increase grip time and impulse without increasing peak force, as does caffeine^7^ or SCH58261 ^8^ delivered directly into the striatum. The grip force task therefore does not report a single quantity; it separates the capacity to sustain output from the force generated at its onset.

The endocannabinoid system is a second candidate for such control, and the two receptor subtypes present very different problems. CB1 receptors are abundant in cortex, basal ganglia, hippocampus and cerebellum, are highly expressed by medium spiny neurons of the dorsal and ventral striatum, are located predominantly on axon terminals, and couple to Gi and Go proteins.^9^ That canonical account has been complicated by evidence of coupling beyond Gi/o and of non-presynaptic localization,^10^ but its functional consequence in striatum is well established: CB1 activation suppresses glutamate release at corticostriatal terminals and also regulates GABA release within the striatum.^11^ In the output nuclei of the basal ganglia of the rat, cannabinoids act on both major excitatory and inhibitory inputs, and the motor consequence depends on which input is active at the time.^12^ The direction of any effect of CB1 blockade is therefore not predictable from receptor distribution.

Nor is it predictable from the plasticity literature. Endocannabinoid-mediated long-term depression at indirect-pathway synapses requires dopamine D2 receptor activation and is absent after dopamine depletion; combining a D2 agonist with an inhibitor of endocannabinoid degradation reduced catalepsy and increased locomotor activity in reserpine- and 6-OHDA-treated mice.^13^ That result was obtained by enhancing endocannabinoid signaling, and whether D2 activation is strictly necessary for the underlying plasticity has itself been questioned.^11^ What blockade of CB1 receptors does in the depleted striatum does not follow from either result and requires direct test.

For CB2 receptors the difficulty is localization rather than direction. Central expression is low and its cellular identity is contested: antibody-independent transcript work finds Cnr2 in a small minority of cortical cells and predominantly in microglia,^14^ a promoter-reporter line shows minimal signal in the naive central nervous system,^15^ and at the protein level most antibodies used in nervous tissue fail validation against tissue lacking the recognized epitope.^16^ A contrary position, arguing for functional neuronal CB2 across several regions, continues to be stated.^17^ What survives the strictest scrutiny nevertheless includes motor structures (the red nucleus and the ventral tegmental area in rodents,^18^ and the pallidal complex in the non-human primate^19,20^ ), and mice lacking Cnr2 in dopamine neurons are hyperactive.^21^ We take up this evidence in full where it bears on the result.

Despite this, the two antagonists have not been compared directly in a monoamine-depleted preparation. We could identify no recent study using intracerebroventricular AM251 or AM630 in reserpine-treated rodents; the nearest comparator is systemic cannabidiol in the progressive low-dose protocol, which, given preventively, attenuates catalepsy and preserves nigral tyrosine hydroxylase labeling.^22^ We therefore asked whether CB1 and CB2 receptor blockade produce different profiles of motor output in reserpine-treated mice, and we tested that difference directly, by a contrast on the force–time balance, rather than by comparing each antagonist against vehicle and inferring a dissociation from the pattern of significance.

## Results and Discussion

### Verification of the Preparation and of Central Delivery

Mice received reserpine at 1 mg/kg 48 and 24 h before testing and displayed the akinetic and rigid state characteristic of the acute regimen. Cannula placement was verified in every animal at the end of the experiment by post-mortem intracerebroventricular infusion of Brilliant Blue dye, examined on coronal faces under a compound light microscope after immersion fixation; distribution of the dye throughout the ventricular system was the criterion for inclusion (Figures 1A and S1). Animals in which the dye did not distribute through the ventricular system were excluded before any behavioral analysis.

**Figure 1:**
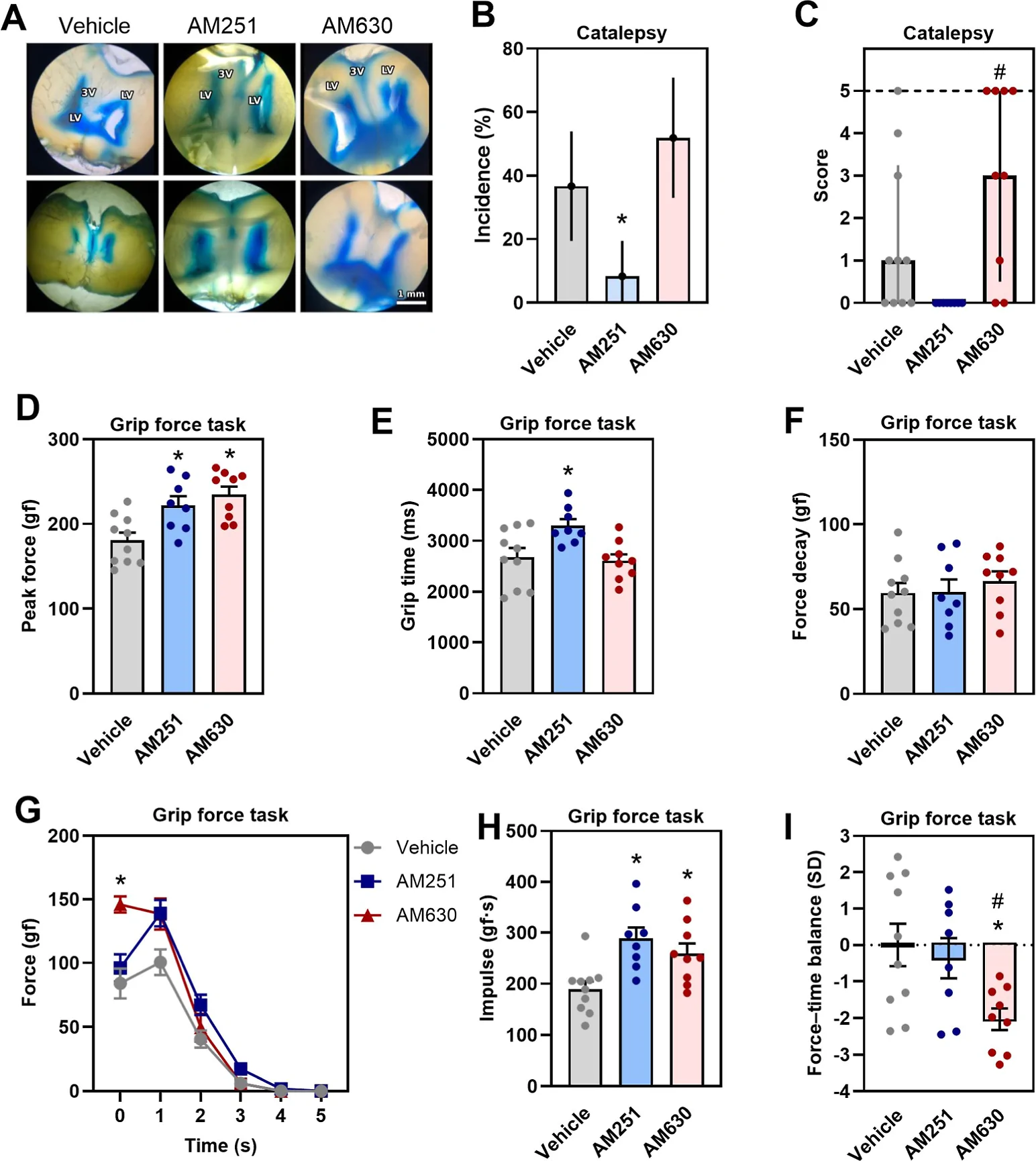
Central blockade of CB1 and CB2 receptors in reserpine-treated mice: catalepsy, grip force and the force–time balance. Adult female Swiss mice received reserpine (1 mg/kg intraperitoneally, 48 and 24 h before testing) followed by a single intracerebroventricular infusion of vehicle (5% DMSO in saline), the CB1 receptor antagonist AM251 (1.0 μg per animal) or the CB2 receptor antagonist AM630 (0.2 μg per animal) 10 min before behavioral testing. *n* = 10 (vehicle), 8 (AM251) and 9 (AM630) throughout. (A) Verification of intracerebroventricular cannula placement: representative images of Brilliant Blue dye distribution through the ventricular system, obtained post mortem under a compound light microscope, two animals per group; the wider set is shown in Figure S1. Ventricular landmarks are labeled in one animal per group, in the upper row: LV, lateral ventricle; 3 V, third ventricle. Scale bar, 1 mm; all frames are at the same magnification. (B) Catalepsy incidence, the percentage of trials in which the imposed posture was maintained, with 95% confidence intervals; analyzed by generalized estimating equations with the animal as the clustering unit, the Mancl–DeRouen small-sample variance correction and test statistics referred to *t*(24). (C) Catalepsy severity on the 0–5 ordinal scale of Shiozaki et al., as median with interquartile range and individual animals; the dashed line marks a score of 5, the ceiling of the scale. Analyzed by the Kruskal–Wallis test followed by Dunn’s test with Holm correction. The # marks the only significant contrast, which is between the two antagonists; neither differs from vehicle. (D) Peak force, (E) grip time and (F) force decay in the grip force task, as mean ± SEM with individual animals; analyzed by one-way analysis of variance followed by Dunnett’s test against vehicle. (G) Force averaged over 1 s intervals from task onset to 5 s, as mean ± SEM; analyzed by repeated-measures analysis of variance with the Greenhouse–Geisser correction, with individual time points compared against vehicle by Welch’s *t*-test and corrected by the Šidák method across the 12 comparisons. (H) Impulse, the integral of force over the trial, as mean ± SEM with individual animals; analyzed as in D–F. (I) Force–time balance: the grip time and peak force of each animal standardized against the vehicle group and subtracted, so that a negative value denotes a shift toward force. The dotted line marks the vehicle balance, which is zero by construction. Groups were compared by one-way analysis of variance and pairs by Welch’s *t*-test. Symbol color identifies the group throughout: gray, vehicle; blue, AM251; red, AM630. *P < 0.05 versus vehicle; ^#^ *P* < 0.05 versus AM251. Error bars are SEM except in B, where they are 95% confidence intervals, and in C, where they are the interquartile range. Estimates, confidence intervals and both uncorrected and Holm-adjusted *P* values for the ten comparisons against vehicle are given in Table S1.

### CB1 Receptor Blockade Reduces the Odds of a Cataleptic Response

Catalepsy was assessed in three trials per animal, and the two aspects of the measurement were analyzed separately, because whether a cataleptic posture occurs and how severe it is carry different information and are subject to different statistical constraints.

Cataleptic postures occurred in 11 of 30 trials in vehicle-treated mice (36.7%), in 14 of 27 trials after AM630 (51.9%) and in 2 of 24 trials after AM251 (8.3%). The three trials contributed by an animal are not independent observations, and treating them as such inflates the effective sample size; incidence was therefore modeled by generalized estimating equations with the animal as the clustering unit, an exchangeable working correlation, the Mancl–DeRouen small-sample variance correction and test statistics referred to *t*(*K* – 3).^23^ CB1 receptor blockade reduced the odds of a cataleptic response (AM251 versus vehicle, OR 0.16, 95% CI 0.03–0.89, *P* = 0.038), whereas the effect of CB2 receptor blockade was not resolved (AM630 versus vehicle, OR 1.86, 95% CI 0.50–6.97, *P* = 0.34), an interval that spans a halving and a sevenfold increase and therefore excludes neither (Figure 1B). The effect should be read as a reduction rather than as a suppression of catalepsy: two of the eight animals in that group did display a cataleptic posture, each on one trial.

Severity, scored 0–5 from the combined durations of forelimb and hindlimb immobility,^24^ differed across groups (Kruskal–Wallis H(2) = 10.68, *P* = 0.005, ε^2^ = 0.41; medians [IQR]: vehicle 1 [0–2.5], AM251 0 [0–0], AM630 3 [1–5]) (Figure 1C). The omnibus difference is carried by the separation between the two antagonists rather than by either of them against vehicle: in Dunn’s test with Holm correction, AM251 differed from AM630 (*P* = 0.003), while neither AM251 (*P* = 0.079) nor AM630 (*P* = 0.19) differed from vehicle. We report this as it stands. The ordinal score summarizes a graded posture and is less sensitive at this sample size than the binary incidence measure, and the vehicle group is itself heterogeneous (individual scores span the full 0–5 range within it), which is characteristic of the acute reserpine preparation and limits what a rank test can resolve against it. The severity data are therefore consistent with the incidence result in direction, and add no independent support to it.

One feature of these data deserves to be stated rather than left for the reader to notice. On both catalepsy measures AM630 moved in the opposite direction to AM251: cataleptic postures occurred in 51.9% of AM630 trials against 36.7% of vehicle trials, and the median severity score was 3 [1–5] against 1 [0–2.5]. Neither difference is resolved against vehicle: the odds ratio for incidence spans a sevenfold increase (1.86, 95% CI 0.50–6.97) and the severity contrast does not survive correction. These numbers are therefore not evidence that CB2 receptor blockade worsens catalepsy. What they do establish is that the separation between the two antagonists, which is the one significant severity contrast, is not carried by AM251 alone.

### Both Antagonists Increase the Force Generated at Task Onset, and by a Similar Amount

In the grip force task, peak force differed between groups (180.9 ± 28.2, 222.0 ± 30.9 and 234.7 ± 28.1 gf for vehicle, AM251 and AM630; *F*(2,24) = 9.01, *P* = 0.001) (Figure 1D). Relative to vehicle, AM630 increased peak force by 53.9 gf (95% CI 22.5–85.3; *P* = 0.0009 versus vehicle, Dunnett; Hedges’ *g* = 1.83, 95% CI 0.77–2.88) and AM251 by 41.2 gf (95% CI 8.8–73.6; *P* = 0.012; *g* = 1.33, 95% CI 0.33–2.33). The two increases are of the same order, their intervals overlap across almost their whole length, and nothing in these data separates the receptor subtypes on this outcome. Force generation at the onset of effort is therefore the component the two manipulations share, not the one that distinguishes them.

### Only CB1 Receptor Blockade Extends the Duration of Maintained Output

Grip time, the total duration over which grip was maintained within the trial and the functional index of the capacity to sustain motor output in this task,^7^ also differed between groups (2681 ± 566, 3300 ± 362 and 2611 ± 375 ms for vehicle, AM251 and AM630; *F*(2,24) = 5.90, *P* = 0.008) (Figure 1E). AM251 increased grip time by 619 ms relative to vehicle (95% CI 113–1125; *P* = 0.016; *g* = 1.21, 95% CI 0.23–2.19), whereas AM630 did not (−70 ms, 95% CI −560 to +421; *P* = 0.92; *g* = −0.14, 95% CI −1.00 to 0.72). This is the outcome on which the two antagonists part company, and the direction of the AM630 estimate is negative rather than merely unresolved.

Force decay, the within-trial decline of force from its peak, did not differ between groups (59.5 ± 18.6, 60.2 ± 20.7 and 66.6 ± 17.2 gf for vehicle, AM251 and AM630; *F*(2,24) = 0.39, *P* = 0.68) (Figure 1F). The intervals are wide (AM251 −20.3 to +21.7 and AM630 −13.3 to +27.4 gf), so this is an unresolved comparison and not a demonstration of equivalence. We report it as a descriptive parameter that did not change detectably and draw no inference from it about the site at which fatigue arises. Separating central from peripheral contributions to a decline in voluntary force requires stimulation of the nerve or muscle, or interpolated maximal contractions; the decline measured during a voluntary contraction is easy to quantify but does not identify the mechanisms that produce it, and a central contribution is present in intact animals as a matter of course.^25^ The case for a central site of action in the present study rests instead on the route of administration: the antagonists were delivered into the lateral ventricle, and their primary access is to central structures.

### The Two Antagonists Act at Different Points in the Trial

The time-resolved record separates when within the trial each effect appears. Force was sampled at 1 s intervals from task onset (*t* = 0 s) to 5 s. It rose to a maximum within the first second and returned to near zero by 4 s in every group (repeated-measures analysis of variance, effect of time *F*(5,120) = 198.9, *P* < 0.0001 with Greenhouse–Geisser correction, ε = 0.53), and the two antagonists altered the shape of that trajectory differently (time × treatment *F*(10,120) = 4.53, *P* = 0.001) (Figure 1G). AM630 raised force at task onset (140.5 ± 21.7 versus 85.0 ± 40.8 gf at *t* = 0 s; *P* = 0.025, Šidák-corrected across the 12 comparisons against vehicle, each tested against the error term of its own time point), the only individual contrast to survive that correction. AM251 sustained force later in the trial, most visibly at 3 s (18.0 ± 8.2 versus 6.2 ± 8.4 gf; *P* = 0.101). The position of the maximum already differs between groups: force was highest at 1 s in vehicle and AM251 animals and at task onset in AM630 animals. These values are not comparable in magnitude with peak force, which is the maximum of the continuous record within a trial, whereas the time-resolved values are forces averaged within 1 s bins and then across animals; both operations attenuate a short-lived maximum reached at an animal-specific instant. The two effects therefore differ in evidential status as well as in timing: the CB2 effect at onset is supported by a surviving pairwise contrast, whereas the CB1 effect on the later trajectory is carried by the interaction term and by grip time, and by no single time point on its own.

Impulse, the integral of force over the trial in gf·s, was increased by both antagonists (189.5 ± 48.3, 288.4 ± 61.9 and 259.2 ± 59.9 gf·s for vehicle, AM251 and AM630; *F*(2,24) = 7.43, *P* = 0.003; AM251 + 98.8, 95% CI 35.7–161.9, *P* = 0.002, *g* = 1.72; AM630 + 69.6, 95% CI 8.5–130.7, *P* = 0.024, *g* = 1.23) (Figure 1H). Impulse is not an independent outcome. Recomputed from the time-resolved force record by trapezoidal integration, it reproduces the reported values exactly (*r* = 1.000, maximum absolute difference below 10^–6^), and we therefore treat it and the time course as one result rather than two. Its interest is that it is the quantity in which the two antagonists agree most closely: total mechanical output rose in both groups, while the way that output was produced did not.

### The Dissociation, Tested Directly

The comparison this study is designed to make is not between each antagonist and vehicle but between the profiles of the two antagonists, and a difference between profiles cannot be established by observing that one comparison is significant and another is not. We therefore standardized peak force and grip time against the vehicle group and tested the contrast between them, the force–time balance, as a single outcome. The balance differed across groups (*F*(2,24) = 4.81, *P* = 0.018) (Figure 1I). AM630 shifted it toward force (−2.03 SD, 95% CI −3.44 to −0.63; *P* = 0.008), whereas the AM251 estimate was not resolved against vehicle (−0.36 SD, 95% CI −2.07 to +1.34; *P* = 0.66), an interval wide enough that a shift of the size seen with AM630 is not excluded for AM251 either. AM630 was displaced 1.67 SD further toward force than AM251 (95% CI 0.29–3.05; *P* = 0.022, Welch).

The balance is defined along an axis on which the vehicle group already varies: within that group, peak force and grip time are inversely correlated, so that animals pulling harder release sooner. A shift along that axis is therefore a shift in a relation the preparation already exhibits. That structure is preserved under CB1 receptor blockade and reversed under CB2 receptor blockade, so what CB2 receptor blockade changes is the relation between the two components rather than the level of one of them alone. The correlations within each group, and the comparisons between them, are reported in Figure S2.

This constrains the interpretation more tightly than the separate comparisons do, and it says something different from what the separate comparisons suggest. Both antagonists raised peak force, by amounts that these data cannot distinguish. What separates them is duration: AM251 raised grip time and AM630 did not, so that in AM251 animals the two components rose together and the balance between them was preserved, whereas in AM630 animals force rose alone and the balance moved. The dissociation is therefore in the duration of effort rather than in its force, and the shift of the balance under CB2 receptor blockade follows from the absence of the grip time effect rather than from a selective action on force. We emphasize the distinction because the pattern of significance against vehicle would, on its own, have suggested the opposite reading, and only the tested contrast decides between them.

### Multiplicity, and What This Cohort Does Not Resolve

Ten comparisons against vehicle were performed across the four grip outcomes and catalepsy incidence, of which five reached nominal significance. Under Holm correction across the full family of ten, two survive: the effect of AM630 on peak force (adjusted *P* = 0.009) and the effect of AM251 on impulse (0.020). The effect of AM251 on peak force (0.095), the effect of AM251 on grip time (0.108) and the effect of AM251 on catalepsy incidence (0.188) do not. We report corrected and uncorrected values together rather than choosing between them. The force–time balance contrast is specified as the primary test of the dissociation rather than a member of that family; it was not, however, pre-registered, and we report it as a planned analysis of an existing dataset. No a priori sample-size calculation was performed. With the group sizes obtained, a two-group comparison has 80% power to detect a standardized difference of *d* = 1.42 and 90% power at *d* = 1.64, so this study addresses large effects and is uninformative about smaller ones; the non-significant comparisons should be read accordingly. Effect estimates with 95% and 90% confidence intervals, together with uncorrected and Holm-adjusted *P* values, are reported for all ten comparisons in Table S1.

One AM251 animal was excluded after testing on a ground that is not among the two criteria set in advance, and we state it here rather than in a footnote. The laboratory record for that animal shows that the intracerebroventricular infusion was given 24 h later than the protocol specifies, so the animal was not tested under the pharmacological condition the group is defined by. We excluded it on that ground and not on its result, but we knew its result when we decided, and the reader is entitled to both analyses. Retaining it returns the cohort to 28 animals and changes the picture in one direction only: the increase in peak force after CB1 receptor blockade falls from *P* = 0.012 to 0.088 and is no longer resolved, while the effect on grip time strengthens (0.008), the effect on impulse weakens (0.009), the reduction in the odds of a cataleptic response strengthens (0.026) and the two balance contrasts strengthen (AM630 versus vehicle 0.008; AM251 versus AM630 0.012). The direction of every estimate is preserved. The claim that would change is the one made here: with that animal retained, the increase in peak force after CB1 receptor blockade is not resolved, and the shared force effect on which this account rests would be carried by CB2 receptor blockade alone. A second animal, in the vehicle group, received its infusion manually after a pump failure and is retained throughout, since the substance delivered was the vehicle and the dose is not the variable.

### Relationship to Dopaminergic Signaling

Reserpine depletes vesicular monoamines by inhibiting VMAT2, so dopaminergic transmission is minimal in this preparation by construction. That constrains the interpretation usefully: an effect of CB1 receptor blockade obtained under these conditions cannot plausibly be explained by restoration of dopaminergic transmission and must arise downstream of it. The relevant question is not whether cannabinoid antagonism restores dopamine signaling but what it does to the circuits that dopamine normally modulates.

Two features of CB1 receptor distribution bear on this. CB1 receptors are located presynaptically on corticostriatal glutamatergic terminals, where their activation suppresses glutamate release, and also on GABAergic terminals within the striatum, where it suppresses inhibitory transmission.^9,11^ Because blockade removes both forms of presynaptic inhibition at once, the net effect on medium spiny neuron output is not predictable from anatomy alone, which is why the direction of the behavioral effect is informative rather than confirmatory. The same indeterminacy holds downstream: in the output nuclei of the basal ganglia, cannabinoids act on both the major excitatory and the major inhibitory inputs, and the motor consequence depends on which input is active at the time.^12^

This bears on an apparent tension with the work of Kreitzer and Malenka,^13^ who improved parkinsonian motor deficits by enhancing endocannabinoid signaling, whereas we increased motor output by blocking CB1 receptors. Their preparation is close to ours (reserpine at 1 mg/kg intraperitoneally, tested 18–24 h later, with catalepsy measured on a horizontal bar), which makes the comparison worth making carefully. The two manipulations are not one mechanism running in opposite directions. Their intervention restored endocannabinoid-mediated long-term depression at indirect-pathway synapses, a plasticity that in their account requires concurrent dopamine D2 receptor activation and is therefore absent after depletion; consistent with that account, the CB1-side manipulation alone (URB597) was without behavioral effect, the D2 agonist alone (quinpirole) reduced catalepsy but did not increase locomotion, and only the combination produced both. We note that the strict necessity of D2 activation for this plasticity has itself been questioned, with D2 receptors described as modulating rather than being absolutely required for LTD induction,^11^ and that the two accounts disagree on this point. In the reserpinized striatum, at any rate, that plasticity is not available to be recruited. What remains is tonic presynaptic control of transmitter release, operating moment to moment rather than as a lasting synaptic change, and it is this component that CB1 receptor blockade removes. One pharmacological qualification belongs with that account, and with the CB2 account below. AM630 is classed as a CB2 receptor inverse agonist rather than a neutral antagonist, and is used in both roles in the literature;^16^ AM251 is likewise an inverse agonist at CB1 receptors. Both compounds therefore reduce constitutive, ligand-independent receptor activity as well as displacing endogenous ligand, so an effect obtained with either does not require that an endocannabinoid tone was present to be blocked: it is equally compatible with a reduction of receptor activity that was never ligand-driven. These data cannot separate the two, and the reading offered here, removal of a tonic brake, is the more restrictive of them. Enhancing a D2-dependent plasticity and removing a tonic brake are distinct interventions, and there is no requirement that they act in the same direction.

### The Cellular Substrate of the CB2 Effect

The identity of the cells expressing CB2 receptors in the central nervous system remains contested, and the evidence supports a more specific statement than either extreme. Transcript-level work using antibody-independent methods places Cnr2 expression at very low abundance (detectable in fewer than 1% of cortical cells, distributed across microglia, neurons and astrocytes, with microglia showing a proportion of positive cells approximately 20-fold greater than neurons and tenfold greater than astrocytes^14^), and a Cnr2-promoter reporter line shows minimal signal in the naive central nervous system, rising under inflammatory conditions.^15^ At the protein level the difficulty has been antibody specificity: of twenty-two antibodies used to detect CB2 receptors in nervous tissue, only four reduce their signal in mice lacking the sequence encoding the recognized epitope, and only two mouse lines with partial Cnr2 deletion exist against which to test them.^16^ The regions in which those four antibodies detect CB2 receptor protein include hippocampus, brainstem and retina, and among them two midbrain structures of direct motor relevance: the red nucleus, where labeling is confined to tyrosine hydroxylase-positive neurons, and the ventral tegmental area, where neurons and astrocytes are both labeled.^16^ Independent work combining in situ hybridization with immunostaining (present in wild-type and CB1-knockout tissue, absent in CB2-knockout tissue) localizes CB2 receptors to magnocellular glutamatergic neurons of the red nucleus, where a CB2 agonist inhibits firing in a dose-dependent manner.^18^ In the non-human primate, an antibody-independent approach reaches the same class of structure: in situ hybridization with riboprobes transcribed from a CB2 sequence cloned from *Macaca fascicularis* spleen RNA, with sense-probe controls, places the strongest CB2 transcript signal in the brain within the internal and external segments of the globus pallidus, in cells identified as neurons by NeuN and parvalbumin co-labeling; laser-microdissected tissue confirmed the signal by PCR.^19^ In identified pallidothalamic projection neurons of the same species, CB1 and CB2 transcripts are co-expressed in essentially every retrogradely labeled cell, with proximity-ligation evidence for CB1–CB2 heteromers; CB2 transcript was unchanged by MPTP parkinsonism itself and fell only after chronic levodopa treatment, whereas CB1 transcript was already slightly raised in parkinsonian animals.^20^ Three limitations belong with this evidence: in situ hybridization cannot resolve pre- from post-synaptic localization, the heteromer detection rests on an unvalidated commercial CB2 antibody and is reported qualitatively, and these primate studies come from a single collaboration.

This offers a plausible substrate for the effect we observed without requiring us to claim it. If CB2 receptor activation suppresses the activity of a midbrain motor structure, blockade would release it, which is consistent in direction with the increase in peak force produced by AM630; conditional deletion of Cnr2 from midbrain dopamine neurons likewise yields a hyperactive phenotype in the open field, in both sexes.^21^ One qualification belongs with that inference: in the study reporting the agonist effect, AM630 alone altered neither red nucleus firing nor motor behavior in naive animals,^18^ which is what would be expected if the endocannabinoid tone available to be blocked emerges only under monoamine depletion, but which remains a difference between preparations that we cannot resolve here. These comparisons differ from ours in age, readout and mode of intervention, and our data cannot localize the effect or assign it to a neuronal or a glial substrate. We report the convergence in direction and leave the substrate open.

That AM630 raised peak force while showing no reduction in catalepsy is worth holding onto rather than explaining away. The two measures are not two windows onto one quantity. Catalepsy scores the maintenance of an externally imposed posture over minutes, and a low score requires the animal to initiate a correcting movement; the grip force task measures a maximal effort recruited over seconds against a pull the animal resists. A manipulation that increases the force available at the onset of an effort need not change the tendency to remain where it has been placed, and the anatomy suggested for the CB2 effect points the same way: releasing a midbrain motor structure would be expected to affect force production more directly than the basal ganglia output that governs postural maintenance. The claim we make from these data is accordingly narrow. Both antagonists raised the force generated at task onset and the total mechanical output of the task, and only CB1 receptor blockade also extended the duration of effort and reduced the odds of a cataleptic response.

### The Grip Force Signature Distinguishes CB2 Blockade from Adenosinergic Manipulation

The task used here discriminates between neuromodulatory interventions, and the most informative comparison available to us is with our own previous work using the same apparatus. In naive mice, systemic caffeine increased grip time and impulse without increasing peak force;^7,8^ the selective adenosine A2A receptor antagonist SCH58261 reproduced that pattern;^8^ and caffeine^7^ or SCH58261 ^8^ delivered directly into the striatum reproduced it again. Systemic dopamine D2 receptor antagonism attenuated the systemic effects while leaving the intrastriatal ones intact, which placed the A2A-D2 interaction largely outside the striatum.^8^ The one previous study of this task in reserpine-treated mice is more restrictive still: across six treatment groups, including l-DOPA, caffeine, haloperidol and their combination, grip time and impulse moved, but no treatment increased peak force.^5^

One feature of the dose is worth stating, because it bears on why an effect was obtained at all. The AM630 dose used here, 0.2 μg per animal, is the lowest of those tested in the study from which it is taken; in that study it did not alter the seizure threshold of healthy mice, while significantly altering it in acutely infected mice, an asymmetry its authors attributed to upregulation or supersensitivity of endocannabinoid signaling in the altered brain.^26^ The reserpine-treated brain is likewise not a healthy one. We do not extend that reasoning to the mechanism, which concerns a different receptor population and a different readout, but it offers a reason why a dose at the bottom of the published range was sufficient here.

Against that background, neither antagonist reproduces the adenosinergic pattern, and that is the finding this comparison yields. Caffeine and SCH58261 raise grip time and impulse and leave peak force untouched, in naive animals and whether given systemically or into the striatum; and in the one previous study of this task in reserpine-treated mice, across six treatment groups including l-DOPA, caffeine, haloperidol and their combination, no treatment increased peak force.^5^ Here both antagonists increased it, by 41 and 54 gf. Force expression is therefore a component of motor output that no adenosinergic or dopaminergic manipulation in this task has been reported to raise, in either preparation, and that blockade of either cannabinoid receptor subtype raises. Three qualifications belong with the comparison. The time-force dissociation was characterized in naive mice, whereas the present study and the reserpine comparison are in the depleted state, and the two preparations do not behave alike: in reserpine-treated mice caffeine alone raised grip time without raising impulse, and a D2 antagonist that attenuates the ergogenic effect in naive animals was there associated with improvement.^5,8^ The caffeine doses also differ between those studies. And the present task engages all four limbs, whereas the earlier studies engaged the forelimbs only, so absolute forces are not comparable across them; the comparison is between profiles rather than between magnitudes.

### The Absence of a Direct Precedent

Recent work on cannabinoid modulation in parkinsonian models has concentrated on CB2 receptor agonism. A selective CB2 agonist reduces apomorphine-induced rotation and nigral dopaminergic loss in 6-hydroxydopamine-lesioned rats,^27^ and another preserves striatal dopamine release and improves anxiety- and depression-related behavior in 6-hydroxydopamine-lesioned mice;^28^ in both, an antagonist appears only as a pharmacological blocking tool rather than as a candidate intervention. Antagonism as an intervention has been comparatively neglected, notwithstanding earlier reports that CB1 receptor antagonists, like agonists, have antidyskinetic activity, a paradox attributed to the presence of CB1 receptors at both excitatory and inhibitory synapses within basal ganglia circuits, and to the pre- and postsynaptic expression of CB1-containing heteromers.^20^ We could identify no recent study measuring striatal dopamine after CB1 or CB2 receptor antagonism given alone, rather than as a tool to block a co-administered agonist, in any model of Parkinson’s disease, and none using intracerebroventricular AM251 or AM630 in reserpine-treated rodents. The nearest comparator in the same model family is systemic cannabidiol in the progressive low-dose protocol, which delays motor alterations, attenuates catalepsy and prevents the loss of tyrosine hydroxylase labeling in the substantia nigra pars compacta;^22^ it differs from the present study in the reserpine regimen, the class of compound, the route of administration and the catalepsy readout (a latency to withdraw the forepaws from a bar against a 180 s ceiling, rather than the ordinal fore- and hindlimb score used here), and so provides context rather than a direct comparison. The design reported here therefore has no close recent precedent, which is a reason to interpret it cautiously and a reason it is worth reporting.

### Limitations

Intracerebroventricular administration establishes that the effects are central in origin but does not localize them: the infused compounds have access to cortical, striatal and brainstem structures, all of which express cannabinoid receptors and all of which contribute to motor output. Cannula placement was confirmed macroscopically by the distribution of Brilliant Blue dye through the ventricular system; no histological verification of the cannula tract was performed, and tissue damage at the injection site was not assessed. Dopamine and its metabolites were not measured in this cohort (the brains were used for placement verification and were not collected for neurochemistry), so the relationship between cannabinoid receptor blockade and dopaminergic signaling is discussed here from the existing literature and is not established by these data. Only one dose of each antagonist was tested, so the effects reported are not characterized as functions of dose; the two doses were also not equimolar (1.8 nmol of AM251 against 0.40 nmol of AM630), so the comparison between compounds is between the doses used rather than between the receptors at matched occupancy. The study includes no non-reserpinized group: the effects are increases in motor output measured on the background of monoamine depletion, and whether they would appear in intact animals is not addressed. Testing order was fixed rather than counterbalanced: the catalepsy test preceded the grip force task. A bar test of up to 3 min immediately before a task that measures sustained force is a plausible source of carry-over, and the grip force results should be read with that order in mind. All animals tested on a given day received the same compound, so treatment is confounded with test day. Only female mice were studied, whereas the previous work in this task from our laboratory used male mice only, so neither body of work extrapolates to the other. The grip task was also run in a four-limb configuration here and in a forelimb-only configuration previously, which precludes comparison of absolute forces. The sample size was not determined in advance and the study resolves only large effects. The within-group correlations between peak force and grip time, reported in Figure S2, rest on 8 to 10 animals each and are correspondingly imprecise; they bear on how the force–time balance should be read and are not an independent result. Animals were tested in four blocks, identifiable in the deposited records by the letter prefix of the animal identifier (A, G, P, V); adding the block to the model for peak force leaves both estimates unchanged (AM251 + 42.1 gf, AM630 + 51.9 gf) and the block term itself is not resolved (*F*(3,21) = 2.16, *P* = 0.12). Body mass did not differ between groups (36.9 ± 2.2, 37.1 ± 3.4 and 37.0 ± 1.5 g for vehicle, AM251 and AM630; *F*(2,24) = 0.02, *P* = 0.98) and did not predict peak force (*r* = 0.22, *P* = 0.27); adjusting peak force for body mass left both effects unchanged (AM251 + 40.5 gf, 95% CI 12.5–68.5, *P* = 0.007; AM630 + 53.6 gf, 95% CI 26.5–80.7, *P* = 0.0005).

### Conclusion

In reserpine-treated mice, blockade of CB1 and of CB2 receptors within the central nervous system both increase the force generated at the onset of effort, by amounts these data cannot distinguish, and both increase the total mechanical output of the task. They separate on duration: CB1 receptor blockade also extends the time over which output is maintained and reduces the odds of a cataleptic response, whereas CB2 receptor blockade does neither, which is why the force–time balance moves under CB2 blockade and not under CB1 blockade. Neither effect is attributable to restored dopaminergic transmission, which reserpine has removed, and neither reproduces the profile that adenosine A2A receptor antagonism produces in the same task, in which force is the one quantity that does not move. The endocannabinoid system therefore contributes to setting the limits of motor output in the depleted state, and the contributions of its two receptor subtypes are separable at the level of behavior: in the duration of effort rather than in its force.

## Methods

### Animals

Adult female Swiss mice (outbred; 8–10 weeks old; 25–45 g) were obtained from the Central Animal Facility of the Federal University of Santa Catarina, Brazil. Animals were housed in groups of up to eight per cage under a 12 h light/dark cycle (lights on at 07:00 h) at 21 ± 1 °C, with ad libitum access to food and water. Cages were enriched with nesting material and polypropylene shelters; shelters were temporarily removed after stereotaxic surgery to prevent interference with the cannula assembly. Animal welfare was monitored daily throughout the experimental period. Only females were studied, and the findings are not generalized to males.

All procedures complied with Brazilian legislation on animal experimentation (Law 11.794/2008 and CONCEA regulations) and were approved by the Animal Ethics Committee of the Federal University of Santa Catarina (CEUA/UFSC, protocol 1069240325). The study is reported in accordance with the ARRIVE guidelines 2.0.

### Experimental Design, Allocation, and Sample Size

The experimental unit was the individual animal. Every animal received reserpine; the design therefore compares cannabinoid receptor antagonists within the monoamine-depleted state and does not include a reserpine-free arm, so it does not test whether the effects are specific to the depleted condition.

Animals were assigned to vehicle, AM251, or AM630 in blocks, with all animals tested on a given day receiving the same compound. No formal randomization procedure was used; animals were taken for a given day in the order in which they came to hand, and the cohort was a single batch of females of the same age, obtained together and housed together throughout under identical conditions of lighting, temperature, feeding and enrichment. Treatment is therefore confounded with test day, and the design does not permit that confound to be separated from the treatment effect; this is stated as a limitation. We report the test block as a covariate in the Limitations, which is the check available to us in place of randomization.

No blinding was used at any stage; the compound given on a test day was known to the experimenter. Peak force, force decay and impulse are extracted by the instrument from the recorded force–time curve and involve no observer judgement. Grip time depends additionally on the tail pull applied by the experimenter, and the catalepsy score on the observers timing of the posture; those two outcomes are not protected in the same way, and we state this rather than assert that the absence of blinding is immaterial to them.

No a priori sample size calculation was performed. At the group sizes obtained (*n* = 8–10), the minimum effect detectable with 80% power at α = 0.05 is *d* = 1.42; non-significant outcomes are therefore reported with their confidence intervals and treated as uninformative rather than as evidence of absence.

Exclusion criteria were defined a priori as incorrect cannula placement or loss of the head-cap assembly before testing. Of 45 animals allocated, 18 were excluded: 11 because post-mortem inspection showed no dye in the ventricular system, indicating incorrect cannula placement, 6 because the head-cap assembly was lost during the post-surgical period, and 1 because the laboratory record shows that its intracerebroventricular infusion was given 24 h later than the protocol specifies, so that it was not tested under the pharmacological condition its group is defined by. The last of these is not one of the two criteria set in advance; it was applied after testing and is reported with the analysis retaining that animal in the Results. 27 animals were analyzed (vehicle, *n* = 10; AM251, *n* = 8; AM630, *n* = 9). One protocol departure occurred among the animals retained: a vehicle animal received its infusion manually because the pump failed. It is retained in every analysis reported here, since the substance delivered was the vehicle and the dose is not the variable. Both departures are flagged in the deposited data. Exclusions were determined before any behavioral analysis and, for cannula placement, on a criterion applied blind to the behavioral result, since placement was verified only after testing. Group sizes are the same for every outcome: vehicle, *n* = 10; AM251, *n* = 8; AM630, *n* = 9 (*N* = 27).

### Reserpine-Induced Parkinsonism

Reserpine (Millipore Sigma-Aldrich Brasil; CAS 50-55-5) was dissolved in 50 μL of glacial acetic acid and diluted in sterile 0.9% NaCl to the final concentration, yielding an injection volume of 1 mL/kg body weight.^29^ Reserpine was administered intraperitoneally at 1 mg/kg in two doses, 48 and 24 h before behavioral testing. Repeated low-dose reserpine, at 0.1 mg/kg every other day, produces a progressive parkinsonian phenotype with face and pharmacological validity in rodents;^3,30^ the acute regimen used here produces instead a rapid and reversible hypokinetic state, suited to symptomatic and mechanistic questions.^2,3^ It follows the dose and route with which this laboratory established the fatigue phenotype under study, in which two intraperitoneal injections of reserpine at 1 mg/kg deplete striatal dopamine and impair both voluntary and forced exercise performance;^4^ the two doses were separated by 24 h here rather than by the 48 h used in that study, so that the depleted state coincided with the day of testing. It is distinct from the repeated low-dose protocol, which produces progressive and cumulative deficits.^30^

### Stereotaxic Surgery

Mice were anesthetized with ketamine (100 mg/kg) and xylazine (7.5 mg/kg), both intraperitoneally, with local lidocaine (0.2%, up to 0.1 mL) at the incision site. A guide cannula was implanted into the right lateral ventricle at the following coordinates relative to bregma: anteroposterior −0.5 mm, mediolateral ±1.0 mm, dorsoventral −2.3 mm.^31^ Behavioral testing took place 9 days after surgery, following 3 days of post-surgical recovery and 3 days of handling and habituation. Analgesia was given pre-operatively as meperidine (20 mg/kg, subcutaneously) and post-operatively as ketoprofen (6 mg/kg, subcutaneously) immediately after surgery and again at 24 and 48 h; enrofloxacin (5 mg/kg, subcutaneously) was given on the same post-operative schedule. The eyes were lubricated with a carbomer gel (Vidisic, Bausch + Lomb) throughout surgery and recovery. During post-operative recovery the animals were monitored by body weight and by water and food intake, and by coat condition, posture and behavior.

### Preparation of AM251 and AM630

Both antagonists were obtained from Cayman Chemical (AM251, CAS 183232-66-8; AM630, CAS 164178-33-0) and prepared as single-use aliquots.

#### AM251

A 5 mg vial was dissolved in a total of 500 μL of anhydrous DMSO added in two steps (200 μL, then 300 μL), with vortexing and sonication at 37 °C until optically clear, giving a stock of 10 mg/mL (10 μg/μL). The stock was divided into 20 μL single-use aliquots, sealed, protected from light, and stored upright at −20 °C. On the day of use, one aliquot was diluted with sterile 0.9% NaCl to a final volume of 400 μL, giving a working solution of 0.5 μg/μL in 5% DMSO.

#### AM630

A 5 mg vial was dissolved in a total of 1.7 mL of anhydrous DMSO, giving a stock of 2.94 mg/mL (2.94 μg/μL); this concentration is set by the solubility of the compound. The stock was aliquoted and stored as above. On the day of use, the working solution was prepared by dilution in sterile 0.9% NaCl to a final volume of 400 μL, giving 0.1 μg/μL. At that concentration the DMSO fraction is 3.4%, not the 5% of the AM251 working solution.

Working solutions were sonicated until clear, kept on ice, protected from light, and used within 24 h. The vehicle group received 5% DMSO in sterile 0.9% NaCl without active compound, matching the higher of the two working solutions; vehicle animals therefore received slightly more DMSO than AM630 animals, not less.

The doses follow a study in which both antagonists were delivered into the lateral ventricle of mice over a dose range: AM630 at 0.2 μg per animal is the lowest dose used there, and AM251 at 1 μg per animal falls between the 0.2 and 5 μg doses used there.^26^ That study used male NMRI mice of 20–25 g and dissolved both compounds in 50% DMSO; the present experiment used female Swiss mice and 5% DMSO, for the reason given below. The concentrations were reached after two preparatory phases that are not part of the dataset reported here: an initial series in which the vehicle was 50% DMSO, abandoned because of poor tolerance after intracerebroventricular delivery, and a dose-finding series with AM630 at 0.07 μg/μL, which preceded the 0.1 μg/μL used in the experiment reported here. The AM630 concentration was constrained by solubility rather than selected: reaching a higher concentration would have required a DMSO fraction reported to be poorly tolerated after intracerebroventricular administration.

### Intracerebroventricular Infusion

Infusions were delivered at 1 μL/min to a total volume of 2 μL per animal, and the injection needle was left in place for 3 min after the infusion to allow diffusion and minimize reflux. The delivered doses were 1.0 μg (1.8 nmol) of AM251 and 0.2 μg (0.40 nmol) of AM630. The two compounds were therefore not administered at equimolar doses, and this is stated as a limitation on the comparison between receptor subtypes.

Behavioral testing began 10 min after the end of the infusion, in a fixed order: catalepsy, then the grip force task. The outcomes reported in this study are catalepsy and neuromuscular performance.

Correct ventricular targeting was verified in every animal after euthanasia by intracerebroventricular infusion of Brilliant Blue dye. Brains were collected following the procedure of Parkinson et al.:^32^ with the animal in ventral recumbency, the acrylic support holding the cannula was removed and the skull was opened with scissors through the stereotaxic openings until the brain was exposed; the brain was then separated from the surrounding tissue with curved forceps passed along its outer surface and beneath it, from the olfactory lobes toward the cerebellum, and any connective tissue or nerve preventing its removal was severed. Brains were fixed in 2% formalin for 24 h and divided in the coronal plane, and the distribution of dye within the ventricular system was inspected on the cut faces under a compound light microscope (Firstlab FL21-BA, 4× objective, ×40 total magnification). No embedding, microtome or cryostat sectioning, histological staining or histopathological assessment was performed. Images were acquired through the eyepiece; with the WF10X eyepiece of this instrument (field number 18 mm) and the 4× objective, the field of view is 4.5 mm in diameter, and the scale bars in Figures 1A and S1 are derived from that value. Animals in which dye did not distribute through the ventricular system were excluded before analysis.

### Catalepsy

Catalepsy was assessed 24 h after the second reserpine injection using the bar test previously established in our laboratory,^4^ with responses scored on the 0–5 ordinal scale of Shiozaki et al.^24^ A horizontal bar (3 mm diameter, 10 cm wide) was positioned 4.5 cm above the surface. The forelimbs were placed on the bar and the duration of the cataleptic posture was timed; the hindlimbs were then placed on the bar and timed independently. The maximum observation time was 3 min per limb pair.

Responses were scored from 0 to 5 by combining the two durations at the 5 and 10 s thresholds defined by Shiozaki et al.^24^ score 0, posture lasting less than 5 s in both limb pairs; score 1, one limb pair 5–10 s and the other less than 5 s; score 2, forelimbs longer than 10 s and hindlimbs less than 5 s; score 3, both limb pairs 5–10 s, or forelimbs less than 5 s and hindlimbs longer than 10 s; score 4, one limb pair longer than 10 s and the other 5–10 s; score 5, both limb pairs longer than 10 s. A score of 0 therefore denotes a posture shorter than 5 s and does not indicate that the animal was never cataleptic. A score of 5 is the ceiling of the scale: it is reached whenever both limb pairs exceed 10 s and does not discriminate between postures of different duration above that threshold.

A score of 2 did not occur in any animal. It requires forelimb immobility to exceed hindlimb immobility across the 5 and 10 s thresholds, and in this bar configuration the opposite holds: the forepaws are placed on the bar and released first, so hindlimb immobility equaled or exceeded forelimb immobility in every animal in which either was recorded. The absence of that score is a property of the task rather than of the scoring. Each animal was evaluated in three consecutive trials, from which two distinct outcomes were derived: severity, as the ordinal score, and incidence, as the presence or absence of a cataleptic posture in each individual trial. The two use different criteria, and the difference matters for reading them together. A trial was recorded as cataleptic whenever the animal maintained the imposed posture instead of replacing the paws immediately (operationally, a maintained posture of at least 1 s in either limb pair), with no further duration requirement; the 5 and 10 s thresholds define the severity score alone. Incidence and severity therefore ask different questions of the same trial: whether the response occurred at all, and how long it lasted. Trial-level incidence has been used in this laboratory since it was introduced to separate a near-universal cataleptic response from a sporadic one in an MPTP model, where the proportion of cataleptic responses distinguished groups that the severity score did not;^33^ it was analyzed there by chi-square on pooled responses, whereas here the repeated trials are modeled with the animal as the clustering unit. We state the operational criterion explicitly, which that earlier work did not. A trial can therefore be cataleptic in an animal whose severity score is 0, and four animals are of that kind: the posture lasted less than 5 s in both limb pairs, giving a score of 0, and at least one trial was recorded as cataleptic. Severity was scored once per animal from the limb immobility times, not derived from the three trials separately. Trials were separated by 1 min, and the catalepsy test preceded the grip force task on the same day. All animals completed three trials.

### Grip Force Task

Neuromuscular performance was assessed 24 h after the second reserpine injection with a grip strength meter (Bonther, Ribeirão Preto, SP, Brazil; 5 kgf capacity; REF CHHQBQ0215). Animals were positioned on a horizontal grid and allowed to grasp it with all four limbs. This departs deliberately from the forelimb-only configuration used in our previous work with this apparatus: reserpine-treated mice show rigidity and postural instability, and four-limb engagement gives a stable grasp in animals that do not reliably sustain a two-paw hold. The consequence is that absolute peak force values obtained here are not directly comparable with forelimb-only measurements, and comparisons with our earlier work are made between profiles of effect rather than between magnitudes. Each animal was then pulled gently by the tail in the direction opposite to the grid until it released the grid. Four trials of 10 s were performed at 1 min intervals and the three best trials were retained for analysis, following procedures previously established by our group.^7^

Force–time curves were recorded continuously and summarized at 1 s intervals from task onset (*t* = 0 s) to 5 s for the time-resolved analysis. Four parameters were extracted from the continuous record rather than from the 1 s summaries, so that peak force and force decay are not recoverable from the summarized trace deposited with the data: peak force, the maximum force reached; grip time, the interval from grid engagement to release; force decay, the reduction in force from its peak within the trial, in gf, a parameter introduced here and not extracted in our previous work with this apparatus; and impulse, the integral of force over time, in gf·s. Impulse is a summary of the time-resolved trace rather than an independent measurement, and is reported as such.

### Statistical Analysis

Analyses were performed in Python 3.11.15 with NumPy 2.4.4, pandas 3.0.2, SciPy 1.17.1, statsmodels 0.14.6 and scikit-posthocs 0.14.0. The analysis script that generates every value reported here is deposited with the data.

The experimental unit was the animal for every outcome except catalepsy incidence, for which the unit of observation was the trial and the animal was the clustering unit.

#### Grip Force Outcomes

Peak force, grip time, force decay and impulse were compared between groups by one-way analysis of variance, with Dunnett’s test against the vehicle group and simultaneous 95% confidence intervals. Normality was assessed within groups by the Shapiro–Wilk test and homogeneity of variance by Levene’s test; no outcome violated either assumption. Effect sizes are Hedges’ *g* against vehicle with 95% confidence intervals. Because Dunnett’s simultaneous intervals are obtained in SciPy by quasi-Monte Carlo integration, a fixed random seed was used so that the reported bounds are exactly reproducible. Simultaneous 90% intervals were computed in the same way and are reported for all ten comparisons in Table S1.

#### Time-Resolved Force

Force averaged over 1 s intervals from task onset to 5 s was analyzed by repeated-measures analysis of variance, with treatment as a between-subject factor and time as the within-subject factor. Sphericity was addressed by the Greenhouse–Geisser correction; degrees of freedom are reported uncorrected and *P* values are corrected. Individual time points were compared against vehicle by Welch’s *t*-test computed separately within each time point, so that variance was not pooled across time, and the resulting *P* values were corrected by the Šidák method across the 12 comparisons.

#### Force–Time Balance

Peak force and grip time were standardized against the mean and standard deviation of the vehicle group, and the balance was defined as the standardized grip time minus the standardized peak force, so that a negative value denotes a shift toward force. Groups were compared by one-way analysis of variance and by Welch’s *t*-test between pairs. This contrast is the primary test of a differential profile between the two antagonists and is reported separately from the family of comparisons against vehicle. The relation between the two components was examined separately within each group by Pearson correlation, and the correlations were compared between groups by Fisher’s *z* transformation.

#### Catalepsy

Incidence was modeled by generalized estimating equations with a binomial family and logit link, the animal as the clustering unit and an exchangeable working correlation, using the bias-reduced covariance estimator of Mancl and DeRouen for small numbers of clusters;^23^ test statistics were referred to a *t* distribution with *K* – 3 degrees of freedom, where *K* is the number of animals. Severity, an ordinal score, was compared by the Kruskal–Wallis test with epsilon-squared as the effect size, followed by Dunn’s test with Holm correction.

#### Body Mass

Body mass was compared between groups by one-way analysis of variance and related to peak force by Pearson correlation; the effect of treatment on peak force was then re-estimated by analysis of covariance with body mass as covariate.

#### Multiplicity

Ten comparisons against vehicle were performed across the four grip force outcomes and catalepsy incidence. Holm correction was applied across that full family, including the comparisons that did not reach nominal significance, and both corrected and uncorrected values are reported.

#### Sample Size

No a priori sample size calculation was performed. The minimum detectable effect at the group sizes obtained was computed post hoc for a two-group comparison at α = 0.05, and non-significant outcomes are reported with confidence intervals rather than interpreted as evidence of absence.

Values are mean ± standard deviation unless stated otherwise; catalepsy severity is given as median with interquartile range. Two-sided *P* < 0.05 was taken as the threshold for statistical significance.

## Supplementary Material



## Data Availability

All data supporting this study are openly available at the Open Science Framework under the DOI 10.17605/OSF.IO/QYGCS, licensed under Creative Commons Attribution 4.0 International (CC BY 4.0). The deposit contains the trial-level and animal-level catalepsy records, the grip force outcomes, the time-resolved force records, a metadata file, and a documented analysis script that regenerates every value reported in this article, including the assumption checks, the effect sizes and the correction for multiplicity. Every file carries the laboratory identifier of the animal, and the four data files can be joined on it.

## Notes

Figure S1, representative images of Brilliant Blue dye distribution through the ventricular system in each experimental group, together with animals excluded for absent ventricular distribution; Figure S2, the relation between peak force and grip time within each group, with the axis along which the force–time balance is measured; Table S1, effect estimates with 95% and 90% confidence intervals and uncorrected and Holm-adjusted *P* values for the ten comparisons against vehicle (PDF)
