# Supplementary material for: Central Blockade of Cannabinoid CB1 and CB2 Receptors Increases Force Generation in Reserpine-Treated Mice, but Only CB1 Blockade Extends the Duration of Effort
Source: ACS Pharmacol Transl Sci. 2026 Sep 29;9(10):2783–94. doi: 10.1021/acsptsci.6c00206 (PMC13693562; doi:10.1021/acsptsci.6c00206)
Supplement: Supplementary file 1 [file pt6c00206_si_001.pdf]

Supporting Information

Central blockade of cannabinoid CB1 and CB2 receptors increases force generation in reserpine-treated mice, but only CB1 blockade extends the duration of effort

Beatriz Carminati dos Santos,[1] Izabely Guedes da Rosa,[1] Laíze de Freitas Mazzardo,[1] Naiara de Souza Santos,[1] Izabel Feltrin Fabro,[1] Tatyana Nery,[1] Amanda Leite Bastos Pereira,[1] and Aderbal S. Aguiar Jr[1,2,*]

[1] Laboratory of Exercise Biology (LABIOEX), Department of Physical Therapy, Centro de Ciências, Tecnologias e Saúde, Federal University of Santa Catarina, Rua Pedro João Pereira 158, Centro Cívico, Araranguá, SC 88905-120, Brazil

[2] Instituto de Ensino e Pesquisa Remederi, Via Anchieta 1616, Vila Moinho Velho, São Paulo, SP 04246-002, Brazil

* Corresponding author. E-mail: aderbal.aguiar@ufsc.br

Manuscript pt-2026-00206c.R2, *ACS Pharmacology & Translational Science*.

This Supporting Information contains 4 pages, 2 figures and 1 table.

Contents

Figure S1. Verification of intracerebroventricular cannula placement across the cohort.

Figure S2. The relation between peak force and grip time within each group.

Table S1. Effect estimates for the ten comparisons against vehicle, with 95% and 90% confidence intervals and uncorrected and Holm-adjusted *P* values.

Figure S2 and Table S1 were generated from the deposited dataset by the deposited analysis script; Figure S1 is a montage of the placement-verification images acquired in the laboratory. Data and code are openly available at the Open Science Framework under the DOI 10.17605/OSF.IO/QYGCS.

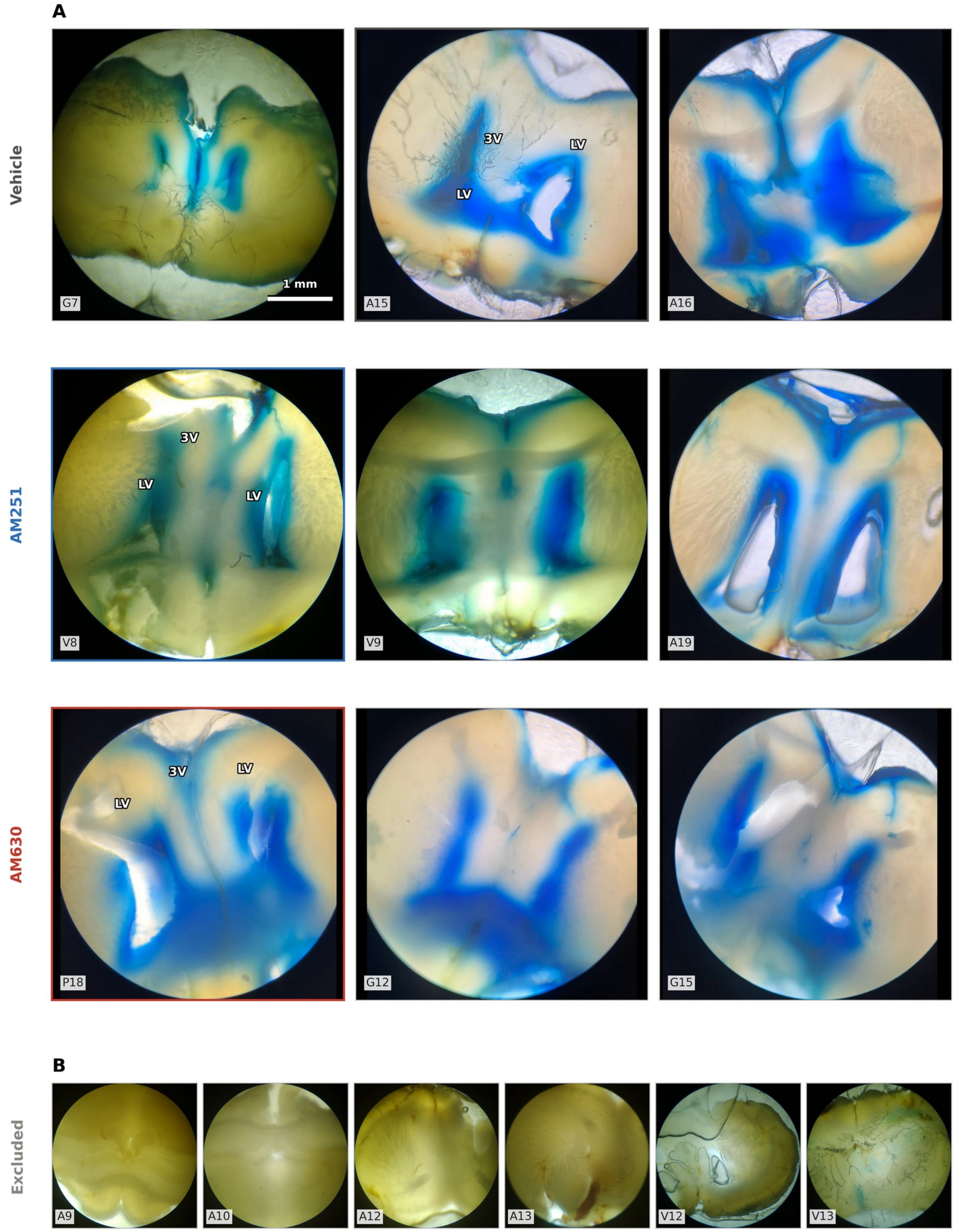

Figure S1. Verification of intracerebroventricular cannula placement across the cohort. Each animal received a post-mortem intracerebroventricular infusion of Brilliant Blue dye, and the coronal cut faces were inspected as described in the Methods. **(A)** Three animals from each group. Distribution of dye through the ventricular system was the criterion for inclusion and was present in every animal entering the analysis (n = 10 vehicle, 8 AM251, 9 AM630). One frame per group, outlined in the group color, carries the ventricular landmarks: LV, lateral ventricle; 3V, third ventricle. **(B)** The six animals excluded before any behavioral analysis because dye did not distribute through the ventricular system. Identifiers are those of the deposited dataset. The circular boundary of each frame is the microscope field of view, 4.5 mm in diameter and identical in every frame. Scale bar, 1 mm.

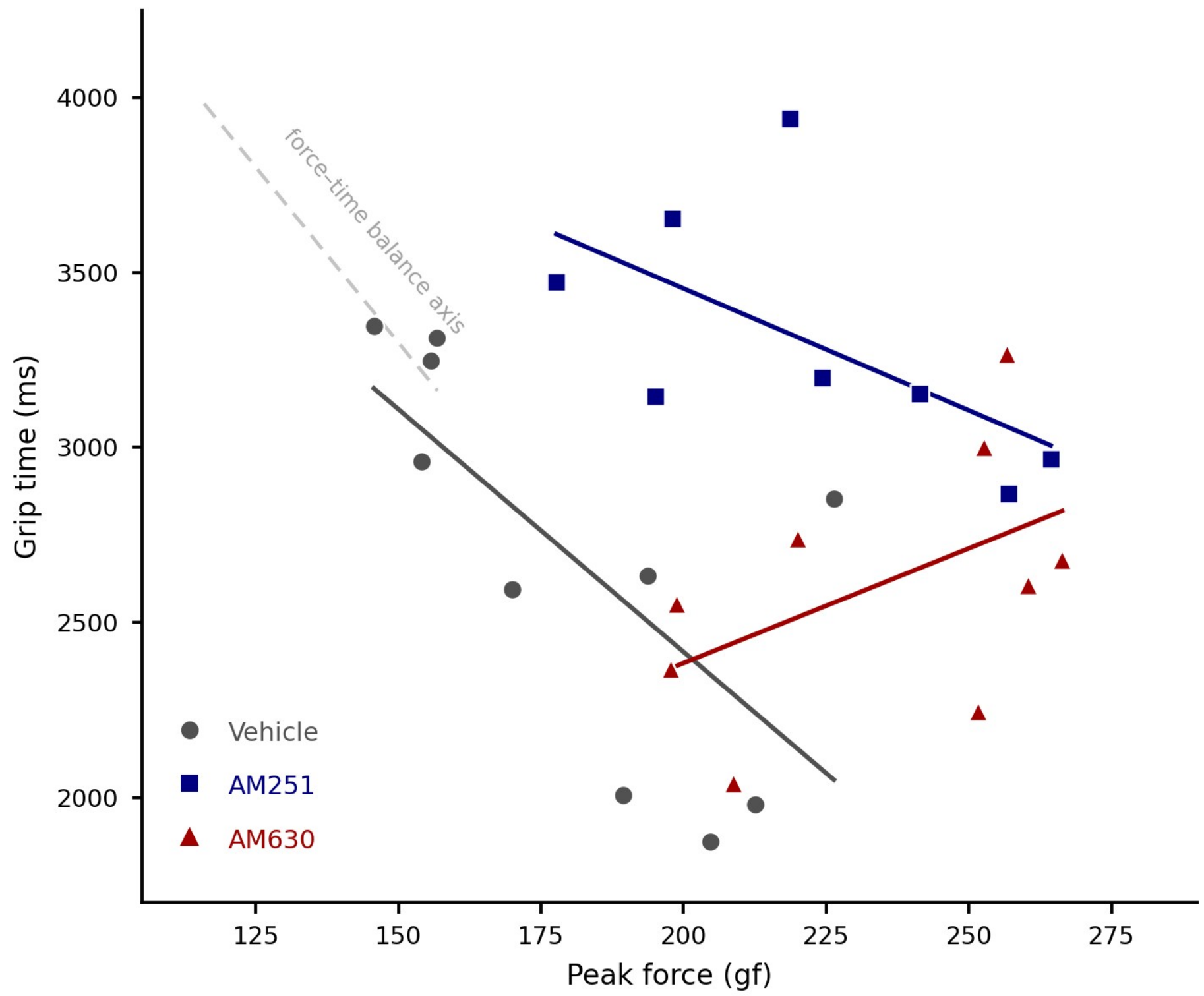

Figure S2. The relation between peak force and grip time within each group. Each symbol is one animal: gray circles, vehicle (n = 10); blue squares, AM251 (n = 8); red triangles, AM630 (n = 9). Solid lines are least-squares fits within each group. Within the vehicle group the two quantities are inversely related (*r* = −0.69, *P* = 0.027), so that animals generating more force release sooner. That structure is preserved under CB1 receptor blockade (*r* = −0.59, *P* = 0.12) and reversed under CB2 receptor blockade (*r* = +0.49, *P* = 0.18). Neither drug correlation is resolved on its own, but the difference between vehicle and AM630 is (Fisher *z* = −2.50, *P* = 0.013), as is the difference between the two antagonists (*z* = −2.02, *P* = 0.044), while vehicle and AM251 do not differ (*P* = 0.77). The dashed line marks the axis along which the force–time balance of Figure 1I is measured: it is the anti-diagonal of the vehicle-standardized space, and therefore the principal axis of individual variation already present under vehicle rather than a dimension introduced by the analysis. The animal excluded for a protocol deviation is not shown. These correlations rest on 8 to 10 animals per group and are correspondingly imprecise; they are reported because they bear on how the force–time balance should be read, and not as an independent result.

Table S1. Effect estimates for the ten comparisons against vehicle, with 95% and 90% confidence intervals and uncorrected and Holm-adjusted *P* values.

Outcome	Group	Estimate	95% CI	90% CI	*P*	Holm-adjusted *P*
Peak force (gf)	AM251	+41.2	+8.8 to +73.6	+13.5 to +68.9	0.012	0.095
Peak force (gf)	AM630	+53.9	+22.5 to +85.3	+27.1 to +80.7	0.0009	**0.009**
Grip time (ms)	AM251	+619	+113 to +1125	+187 to +1052	0.016	0.108
Grip time (ms)	AM630	−70	−560 to +421	−489 to +350	0.924	1.000
Force decay (gf)	AM251	+0.7	−20.3 to +21.7	−17.3 to +18.7	0.996	1.000
Force decay (gf)	AM630	+7.0	−13.3 to +27.4	−10.4 to +24.5	0.639	1.000
Impulse (gf·s)	AM251	+98.8	+35.7 to +161.9	+44.9 to +152.8	0.002	**0.020**
Impulse (gf·s)	AM630	+69.6	+8.5 to +130.7	+17.3 to +121.9	0.024	0.147
Catalepsy incidence (odds ratio)	AM251	0.16	0.03 to 0.89	0.04 to 0.66	0.038	0.188
Catalepsy incidence (odds ratio)	AM630	1.86	0.50 to 6.97	0.62 to 5.56	0.342	1.000

Estimates for the four grip force outcomes are differences from vehicle with Dunnett simultaneous intervals; catalepsy incidence is an odds ratio from generalized estimating equations with the animal as the clustering unit, an exchangeable working correlation and the bias-reduced covariance estimator of Mancl and DeRouen, with test statistics referred to a *t* distribution with 24 degrees of freedom. Holm correction is across the family of ten. n = 10 (vehicle), 8 (AM251) and 9 (AM630) throughout. Ninety percent intervals are reported alongside the conventional ones because a 95% interval for an unresolved comparison necessarily contains the null value and therefore restates the result of the test, whereas the 90% interval bounds the range of effects that remain compatible with these data. Bold marks the two comparisons that survive correction.
